# Dynapenic Abdominal Obesity and Disability in Middle-Aged and Older Adults: A Systematic Review and Meta-Analysis of Prospective Cohort Studies

**DOI:** 10.3390/healthcare14142125

**Published:** 2026-07-15

**Authors:** Shih-Sen Lin, Sung-Yun Chen, Shih-Chun Lin, Hsiao-Chi Tsai, Yu-Chen Su, Ya-Fang Chen, Yi-Fan Tsai, Shu-Fang Chang

**Affiliations:** 1 Division of Chest Medicine, Department of Internal Medicine, Shin Kong Wu Ho-Su Memorial Hospital, Taipei City 111045, Taiwan; samlin.skhctc@gmail.com; 2 School of Nursing, College of Nursing, National Taipei University of Nursing and Health Sciences, Taipei City 112303, Taiwan; sue6227@hsc.edu.tw; 3 Nursing Department, Taipei City Hospital Renai Branch, Taipei City 106243, Taiwan; b1717@tpech.gov.tw; 4 Department of Nursing, Dalin Tzu Chi Hospital, Buddhist Tzu Chi Medical Foundation, Chiayi 62247, Taiwan; t780927t@yahoo.com.tw; 5 Outpatient Nursing Department, Cardinal Tien Hospital AN Kang Branch, New Taipei City 231051, Taiwan; 890224@cth.org.tw; 6 Nursing Department, Taipei City Hospital Yangming Branch, Taipei City 11146, Taiwan; b1655@tpech.gov.tw

**Keywords:** disability, dynapenic abdominal obesity, prospective cohort study, systematic review, meta-analysis

## Abstract

**Background:** Dynapenic abdominal obesity (DAO), the coexistence of low muscle strength and abdominal obesity, may accelerate disability. However, its longitudinal association with disability remains unclear. In this study, we examined the relationship between DAO and disability in middle-aged and older adults. **Methods:** A systematic review and random-effects meta-analysis of prospective cohort studies was conducted to examine longitudinal associations following the PRISMA 2020 guidelines (PROSPERO: CRD42024609352). Comprehensive database searches were conducted in PubMed, Embase, MEDLINE (Ovid), CINAHL (EBSCOhost), and the Cochrane Library from database inception through 31 January 2025. To ensure that the review reflected the most recent evidence prior to manuscript submission, an updated search using the same search strategy and eligibility criteria was conducted on 31 May 2026. When multiple effect estimates were reported, the most fully adjusted estimates were selected. Because odds ratios (ORs) and hazard ratios (HRs) are different effect measures and are not directly comparable, they were analyzed separately. A random-effects model was used for quantitative synthesis. **Results:** Five cohort studies (*n* = 33,670; mean follow-up: 5.3 years) were included. Of these, four studies reported adjusted ORs and one reported an HR. DAO was associated with significantly higher odds of disability (pooled OR = 2.13, 95% CI: 1.74–2.60, *I*^2^ = 0%). Subgroup analyses demonstrated significant associations across predominant population types and age groups, with ORs ranging from 2.09 to 2.18. **Conclusions:** DAO is associated with increased odds of future disability across population types and age groups. These findings highlight the importance of early identification of DAO and suggest that interventions targeting muscle strength and abdominal obesity represent promising strategies for disability prevention; however, their effectiveness should be confirmed in future prospective intervention studies.

## 1. Introduction

The World Health Organization reports that global life expectancy continues to rise, contributing to a rapidly aging population worldwide, with the number of individuals aged 60 years and older surpassing that of children younger than 5 years in 2020 and projected to reach 2.1 billion by 2050 [1]. This demographic shift presents a major global health challenge and highlights the need for effective strategies to maintain functional independence in older adults.

Although muscle mass may remain relatively stable with age, muscle strength declines progressively, particularly in handgrip strength—a condition referred to as dynapenia [2]. Dynapenia is common among older adults, with reported prevalence rates ranging from 10% to 16.9% across different countries [3,4,5]. Evidence also indicates that dynapenia is associated with adverse outcomes, including increased mortality risk (HR = 1.23, 95% CI: 1.04–1.45) [6].

Concurrently, the prevalence of overweight and obesity continues to rise globally. Abdominal obesity, characterized by the accumulation of visceral adipose tissue, is strongly associated with metabolic disorders, chronic diseases, and increased mortality risk [7,8,9]. Compared with general obesity, central fat accumulation poses a greater threat to metabolic health because of its proximity to vital organs [7]. Evidence suggests that declines in muscle strength and increases in abdominal adiposity may begin in midlife and worsen with aging. These changes may interact and contribute to the development of disability later in life. Dynapenic abdominal obesity (DAO), defined as the coexistence of reduced muscle strength and abdominal obesity, is increasingly recognized as a clinically relevant condition. Its prevalence in older populations has been reported to range from approximately 7.2% to 10.7% [4,6]. DAO has been linked to several adverse health outcomes, including impaired physical function, multimorbidity, and increased mortality [4,10,11,12,13,14,15,16,17].

Although a previous systematic review included disability among several adverse health outcomes associated with DAO [10], disability was not the primary focus of that review. Furthermore, the longitudinal association between DAO and disability has not been comprehensively synthesized using prospective cohort evidence, and potential differences across settings, predominant population types, and age groups remain unclear. In this study, we therefore aimed to systematically review and meta-analyze prospective cohort studies examining the association between DAO and disability among middle-aged and older adults and to explore whether this relationship varies across different populations. By specifically focusing on disability as the primary outcome, we provide a more comprehensive synthesis of the available longitudinal evidence that may offer important implications for nursing practice, disability prevention, and healthy aging strategies.

## 2. Materials and Methods

### 2.1. Research Design

This systematic review and meta-analysis adhered to the updated Preferred Reporting Items for Systematic Reviews and Meta-Analyses (PRISMA) 2020 statement [18] (see the PRISMA 2020 checklist in Supplementary Material Table S1).

### 2.2. Data Sources and Search Strategy

Comprehensive database searches were conducted in PubMed, Embase, MEDLINE (Ovid), CINAHL (EBSCOhost), and the Cochrane Library from database inception through 31 January 2025, in accordance with the PROSPERO-registered protocol (CRD42024609352). To ensure that the review reflected the most recent evidence prior to manuscript submission, an updated search using the same search strategy and eligibility criteria was conducted on 31 May 2026.

The PubMed search strategy combined Medical Subject Headings (MeSH) terms and free-text keywords using Boolean operators as follows: (“dynapenia” OR “muscle weakness” OR “low grip strength” OR “low muscle strength”) AND (“abdominal obesity” OR “central obesity” OR “visceral fat” OR “dynapenic abdominal obesity”) AND (“disability” OR “functional limitation” OR “activities of daily living” OR “instrumental activities of daily living”). Similar search strategies were adapted for Embase, MEDLINE (Ovid), CINAHL (EBSCOhost), and the Cochrane Library according to the indexing terms and syntax requirements of each database. Detailed search strategies for all databases are provided in Table S2. No backward or forward citation searching was performed, and gray literature was not searched. No language restrictions were applied during the literature search. An information specialist or librarian was not consulted in the development of the search strategy. Given the narrowly defined research question, which focused exclusively on prospective cohort studies examining the longitudinal association between dynapenic abdominal obesity and disability in middle-aged and older adults, only a limited number of eligible studies was expected despite the comprehensive database search and the absence of language restrictions.

All records retrieved from the databases were exported to EndNote (Clarivate Analytics) for reference management. Duplicate records were identified using the automatic deduplication function in EndNote based on combinations of title, author name, publication year, and DOI fields. A manual review was subsequently conducted to verify potential duplicates and ensure accurate removal of redundant records. The study selection process followed PRISMA guidelines. Two reviewers (S.-Y.C. and S.-F.C.) independently screened the titles and abstracts of all retrieved records to identify potentially eligible studies. The full texts of potentially relevant studies were then evaluated for eligibility in accordance with predefined inclusion criteria. Disagreements between the reviewers were resolved through discussion or consultation with a third reviewer.

### 2.3. Inclusion and Exclusion Criteria

Eligibility criteria were defined according to the PECO (Population, Exposure, Comparator, Outcome) framework. Population (P): Middle-aged and/or older adults, as defined by the original studies (generally ≥50 years or explicitly described as middle-aged or older populations). Exposure (E): DAO, defined as the coexistence of reduced muscle strength (e.g., low handgrip strength or physical performance) and abdominal obesity (e.g., elevated waist circumference or visceral adiposity). Comparator (C): Individuals without DAO, including those with neither dynapenia nor abdominal obesity or with only one of these conditions. Outcome (O): Disability-related outcomes, including activities of daily living (ADL), instrumental activities of daily living (IADL), mobility limitations, or disability assessed using validated instruments. Only prospective cohort studies were included to ensure temporal relationships between exposure and outcome.

### 2.4. Data Extraction

To minimize potential overlap bias, the source populations and cohort characteristics of all included studies were carefully reviewed. When multiple publications originated from the same cohort, only one eligible effect estimate per outcome was included in the meta-analysis to avoid double counting of participants and maintain data independence. Data extracted from each study included sample size, country, sex distribution, participant age, follow-up duration, DAO criteria, disability criteria, and adjusted covariates. Adjusted covariates were extracted and are summarized in Table S2. In addition, study characteristics, including cohort name, follow-up period, and outcome definition, were carefully compared to ensure data independence. A conservative approach was adopted to minimize potential overlap and avoid double counting of participants by carefully comparing cohort characteristics, follow-up periods, and outcome definitions across studies.

### 2.5. Definition and Standardization of Outcomes

The operational definitions of DAO and disability varied among the included studies. DAO was generally defined as the coexistence of low handgrip strength and abdominal obesity; however, the specific cutoffs differed among the studies. Handgrip strength thresholds ranged from <15 kg to <33 kg for men and from <8 kg to <19 kg for women. Abdominal obesity was primarily defined using waist circumference, with cutoffs that varied by population. For example, in some European cohorts, abdominal obesity was defined as a waist circumference of >100 cm for men and >87 cm for women, whereas in studies in Asian populations, thresholds of ≥90 cm for men and ≥85 cm for women were employed. In several studies, body mass index (BMI) criteria were also incorporated.

Disability outcomes were assessed using several validated instruments, including activities of daily living (ADL), instrumental activities of daily living (IADL), the Barthel Index, self-reported functional limitations, and mobility performance measures such as the 400 m walk test or gait speed <0.8 m/s. Disability was generally defined as dependence in at least one ADL or IADL domain or reduced performance on standardized mobility assessments. The disability instruments, operational definitions, and corresponding cutoff criteria used in each study were extracted and standardized to improve comparability across studies.

### 2.6. Quality Assessment

Two authors (S.-F.C. and S.-Y.C.) independently assessed study quality by using the Newcastle–Ottawa Scale (NOS), a validated tool used to evaluate the risk of bias in nonrandomized studies, particularly cohort studies [19]. The NOS is used to evaluate methodological quality across three domains: selection, comparability, and outcome. The selection domain refers to the representativeness of the study sample, appropriateness of participant selection, and accuracy of exposure measurement. The comparability domain evaluates whether the study groups are comparable and whether potential confounding factors are adequately controlled for. The outcome domain is used to assess the adequacy of outcome measurement, validity of the assessment methods, and completeness of follow-up. For the included prospective cohort studies, the NOS was applied to evaluate comparability, outcome assessment, and exposure, with a maximum possible score of 9. A score of ≥7 indicated a low risk of bias, a score of 4–6 indicated a moderate risk of bias, and a score of <4 indicated a high risk of bias [19].

### 2.7. Statistical Analysis

Statistical analyses were conducted using data extracted from the included cohort studies. When multiple effect estimates were reported, the most fully adjusted estimates were selected to reduce potential confounding. Because odds ratios (ORs) and hazard ratios (HRs) are different effect measures and are not directly comparable, they were analyzed separately. A random-effects model was used for quantitative synthesis.

A random-effects model was applied to account for variation across studies. Heterogeneity was assessed using the chi-square (χ^2^) test and the *I*^2^ statistic, with values of 25%, 50%, and 75% representing low, moderate, and high heterogeneity, respectively [20]. Subgroup analyses were conducted in advance based on predominant population type (Asian vs. non-Asian) and age group (≥65 years vs. <65 years) to explore potential differences across predominant population types and age groups. For age-based subgroup analyses, studies were classified according to the reported mean age or age range of the study population. Studies with a mean age <65 years or age ranges predominantly below 65 years were assigned to the <65 years subgroup, whereas studies with a mean age ≥65 years or populations explicitly defined as older adults were assigned to the ≥65 years subgroup.

Sensitivity analysis was performed using a leave-one-out approach to assess whether any single study had a substantial influence on the overall results [21]. Publication bias was not formally assessed because fewer than 10 studies were included in the meta-analysis, as funnel plots and statistical tests for publication bias are considered unreliable when the number of studies is small. All analyses were conducted using Review Manager (RevMan), version 5.4.

### 2.8. Certainty of Evidence (GRADE)

The certainty of evidence for the association between DAO and disability was assessed using the GRADE approach. The evaluation considered study design, risk of bias, consistency, and precision.

## 3. Results

### 3.1. Study Sample

**Figure 1:**
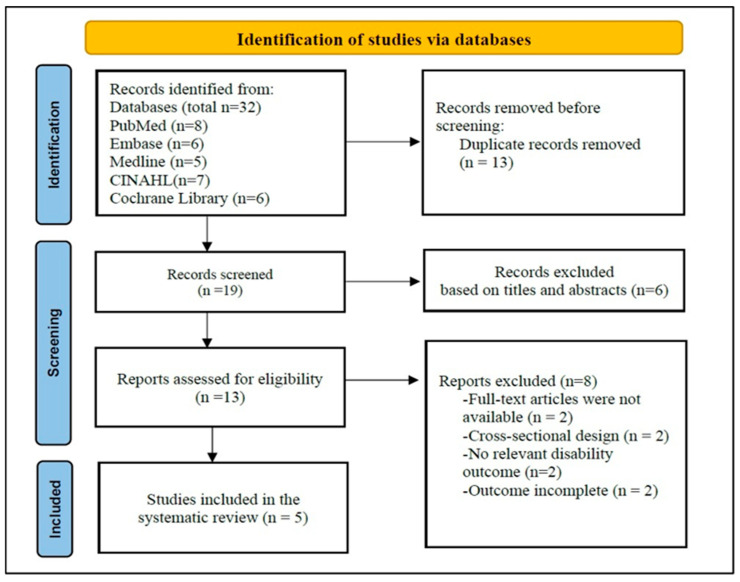
PRISMA 2020 flow diagram for dynapenic abdominal obesity and disability.

The study selection process is presented in Figure 1. A total of 32 records were identified through database searches (PubMed = 8, Embase = 6, MEDLINE = 5, CINAHL = 7, and Cochrane Library = 6). After the removal of 13 duplicate records, 19 records remained for title and abstract screening. Six records were excluded during this stage. The remaining 13 full-text articles were assessed for eligibility. Of these, eight articles were excluded because the full text was unavailable (n = 2), the study employed a cross-sectional design (n = 2), no relevant disability outcome was reported (n = 2), or outcome data were incomplete (n = 2). Ultimately, five prospective cohort studies involving 33,670 participants were included in the systematic review. Four studies reporting adjusted ORs were included in the quantitative meta-analysis, whereas one study reporting a HR was analyzed separately. The main characteristics of the included studies, including country, sample size, age distribution, follow-up duration, DAO criteria, and disability criteria, are summarized in Table 1. Adjusted covariates are presented in Supplementary Table S3.

### 3.2. NOS Quality Assessment

The results of the quality assessment indicated that the overall risk of bias among the included studies ranged from low to high. Three studies received NOS scores between 4 and 6, indicating a moderate risk of bias. These scores primarily reflected limitations in sample representativeness, comparability between study groups, or adequacy of follow-up. One study [15] received a score of 3.5, indicating a high risk of bias because of methodological limitations across several domains. One study [13] received a NOS score of 7, indicating a low risk of bias due to stronger study design, more comprehensive follow-up procedures, and better control of potential confounding factors. Detailed quality assessment results are presented in Table 2.

### 3.3. Association Between DAO and Disability

**Figure 2:**
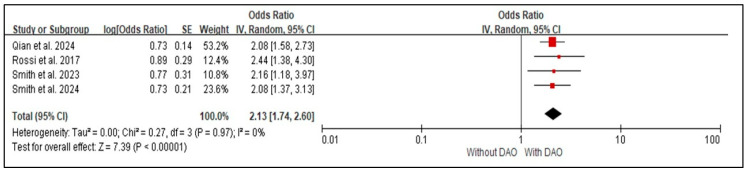
Forest plot of the association between DAO and disability. Pooled ORs and 95% CIs were calculated using a random-effects model. Heterogeneity among studies was assessed using the *I*^2^ statistic. The size of the squares represents the weight assigned to each individual study, while the horizontal lines indicate the 95% confidence intervals. The diamond represents the overall pooled estimate. CI: confidence interval [13,14,16,17].

Four studies were included in the quantitative meta-analysis. The pooled OR for disability among individuals with DAO was 2.13 (95% CI: 1.74–2.60, *p* < 0.00001), indicating a significantly increased likelihood of disability compared with individuals without DAO. One study (Rossi et al. [15]) reported a HR and was therefore excluded from the quantitative synthesis. Nevertheless, the findings of Rossi et al. [15] were directionally consistent with the pooled results, demonstrating a significantly increased risk of disability among participants with DAO during follow-up (HR = 3.39, 95% CI: 1.91–6.02). No heterogeneity was detected across the four included studies (Tau^2^ = 0.00, χ^2^ = 0.27, df = 3, *p* = 0.97, *I*^2^ = 0%) (Figure 2).

### 3.4. Sensitivity Analysis

The leave-one-out sensitivity analysis results demonstrated that the pooled effect estimates remained stable after sequential removal of each study, with pooled ORs ranging from 2.09 to 2.18. These findings indicate that no single study substantially influenced the overall results, supporting the robustness of the association between dynapenic abdominal obesity and disability (Table 3).

### 3.5. Subgroup Analysis

For subgroup analyses based on population type, two studies were classified as involving predominantly Asian populations [13,17], whereas two studies were classified as involving predominantly non-Asian populations [14,16]. For age-based subgroup analyses, two studies included participants aged <65 years [13,16], whereas one study included participants aged ≥65 years [14]. One study [17] could not be classified into either age subgroup because participants spanned both age categories and age-specific estimates were unavailable. Rossi et al. [15] was analyzed separately because it reported a hazard ratio.

### 3.6. Subgroup Analysis by Population Type

**Figure 3:**
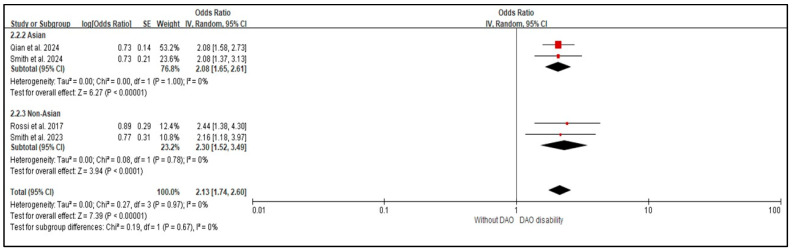
Forest plot of subgroup analysis by predominantly population type (Asian vs. non-Asian populations). Pooled ORs and 95% CIs were calculated using a random-effects model. Heterogeneity was assessed using the *I*^2^ statistic. The size of the squares represents the weight assigned to each individual study, while the horizontal lines indicate the 95% confidence intervals. The diamond represents the overall pooled estimate. CI: confidence interval [13,14,16,17].

Figure 3 presents the subgroup analysis results of the association between DAO and disability according to population type. DAO was significantly associated with disability in predominantly Asian populations (OR = 2.08, 95% CI: 1.65–2.61, *p* < 0.00001), with no observed heterogeneity (Tau^2^ = 0.00, χ^2^ = 0.00, df = 1, *p* = 1, *I*^2^ = 0%), and predominantly non-Asian populations (OR = 2.30, 95% CI: 1.52–3.49, *p* < 0.0001), with no observed heterogeneity (Tau^2^ = 0.00, χ^2^ = 0.08, df = 1, *p* = 0.78, *I*^2^ = 0%). The pooled estimate showed that individuals with DAO had significantly higher odds of disability than those without DAO (OR = 2.13, 95% CI: 1.71–2.60, *p* < 0.00001), with no heterogeneity among the studies (Tau^2^ = 0.00, χ^2^ = 0.27, df = 3, *p* = 0.97, *I*^2^ = 0%). No significant difference was observed between the population type subgroups (χ^2^ = 0.19, df = 1, *p* = 0.67, *I*^2^ = 0%), indicating that the association between DAO and disability was consistent across Asian and non-Asian populations.

### 3.7. Subgroup Analysis by Age Group

**Figure 4:**
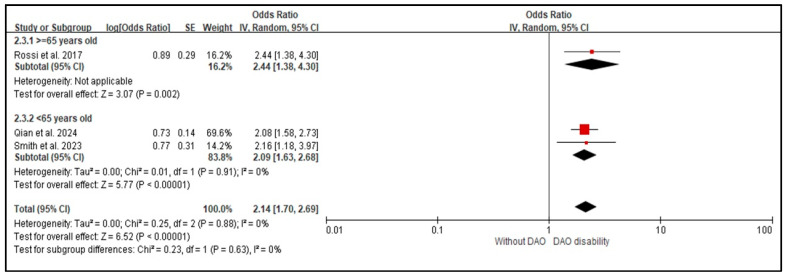
Forest plot of subgroup analysis by age group (≥65 years vs. <65 years). Pooled ORs and 95% CIs were estimated using a random-effects model. Heterogeneity was assessed using the *I*^2^ statistic. The size of the squares represents the weight assigned to each individual study, while the horizontal lines indicate the 95% confidence intervals. The diamond represents the overall pooled estimate. CI: confidence interval [13,14,16].

Because Smith et al. [17] included participants across a broad age range (56–82 years) and did not provide age-stratified estimates, this study could not be classified into either age subgroup and was therefore excluded from the age-based subgroup analysis. Figure 4 presents the subgroup analysis results of the association between DAO and disability by age group. Among adults aged ≥65 years, DAO was significantly associated with higher odds of disability (OR = 2.44, 95% CI: 1.38–4.30, *p* = 0.002). Among adults aged <65 years, DAO was also significantly associated with higher odds of disability (OR = 2.09, 95% CI: 1.63–2.68, *p* < 0.00001). The pooled estimate showed that individuals with DAO had significantly higher odds of disability than those without DAO (OR = 2.14, 95% CI: 1.70–2.69, *p* < 0.00001). No significant difference was observed between the age subgroups (χ^2^ = 0.23, df = 1, *p* = 0.63, *I*^2^ = 0%), indicating that the association between DAO and disability was consistent across age groups.

### 3.8. Certainty of Evidence

The certainty of evidence for the association between dynapenic abdominal obesity and disability was rated as low according to the GRADE approach. Although the included studies demonstrated consistent findings, no statistical heterogeneity (*I*^2^ = 0%), and a relatively large effect size (OR = 2.13), the certainty of evidence remained low because all included studies were observational cohort studies and residual confounding could not be completely excluded. Furthermore, publication bias could not be formally evaluated because fewer than 10 studies were available for analysis (Table 4).

## 4. Discussion

Individuals with DAO had significantly higher odds of disability than those without DAO. The results remained stable in the sensitivity analyses, suggesting that the overall findings were not substantially influenced by any single study. Similar associations were observed across different study settings, population types, and age groups. Taken together, these findings suggest that DAO may help identify middle-aged and older adults who are at greater risk of disability. Given the impact of disability on physical function, quality of life, and healthcare utilization, early identification of DAO may help identify individuals at increased risk of disability and may facilitate the development of promising intervention strategies, although their effectiveness requires further investigation.

DAO, defined as the coexistence of reduced muscle strength and abdominal obesity [4,6], may contribute to disability through several interconnected pathways. Reduced muscle strength can impair mobility, balance, and the ability to perform daily activities, thereby increasing vulnerability to functional decline. Concurrently, abdominal obesity, particularly the accumulation of visceral fat, is associated with chronic low-grade inflammation, insulin resistance, and metabolic dysfunction, all of which may further accelerate the loss of muscle strength and physical performance. The coexistence of these two conditions may therefore have a compounded effect on physical function, resulting in a greater risk of disability than either condition alone. The present findings are consistent with previous studies demonstrating that DAO is associated with adverse health outcomes, including poorer physical function, mobility limitations, disability, and mortality [10,11,12,13,14,15,16,17]. For example, Qian et al. [13], Rossi et al. [14], and Smith et al. [16,17] reported significantly higher disability risk among individuals with DAO. In addition, Rossi et al. [15], which was synthesized narratively because it reported a HR rather than an OR, demonstrated a similar longitudinal association between DAO and disability. Collectively, these findings support the view that DAO represents a vulnerable phenotype characterized by both impaired physical capacity and adverse metabolic burden. The present findings are also supported by evidence from studies examining related body-composition and muscle-function phenotypes. Previous research has shown that reduced muscle strength alone is associated with functional limitation, disability, and mortality in older adults [6]. Likewise, obesity has been linked to impaired mobility, poorer physical performance, and increased risk of adverse health outcomes [7,8,9]. Furthermore, studies examining dynapenia, abdominal obesity, and related phenotypes have suggested that the coexistence of muscle weakness and excess adiposity may have a greater impact on physical function than either condition alone [4,10,16,17]. These observations support the hypothesis that impaired muscle function and metabolic burden may act synergistically to accelerate functional decline and disability. Therefore, DAO may represent a clinically relevant phenotype within a broader spectrum of adverse body-composition conditions associated with disability risk.

All studies included in the present review were conducted among community-dwelling populations, and the association between DAO and disability was consistently observed across studies. Previous studies have reported similar findings. Rossi et al. [14] demonstrated that DAO was associated with worsening disability, whereas Smith et al. [16,17] reported significant associations between DAO and ADL disability in large population-based cohorts. In addition, Máximo et al. [4] found that individuals with DAO exhibited poorer ADL and IADL performance, slower gait speed, and impaired balance compared with those without DAO. Taken together, these findings suggest that the combined effects of reduced muscle strength and abdominal obesity may negatively influence physical function in community-dwelling adults. Therefore, routine assessment of muscle strength and abdominal obesity may be valuable for the early identification of individuals at risk of disability.

Consistent associations were observed across population types. Although the pooled effect estimate was numerically higher in predominantly non-Asian populations, DAO was significantly associated with disability in both predominantly Asian and predominantly non-Asian populations, and no significant subgroup difference was observed. These findings suggest that the adverse effects of reduced muscle strength combined with abdominal obesity may be relevant across diverse populations despite differences in body composition, lifestyle, and healthcare systems [22,23,24]. The population-type subgroup analysis was performed because diagnostic criteria for abdominal obesity differ across ethnic populations. Previous studies have shown that Asian populations tend to accumulate visceral adiposity and develop metabolic complications at lower levels of BMI and waist circumference than Western populations, leading to the recommendation of lower waist circumference cutoffs for Asian populations [25,26,27]. Despite these differences in anthropometric thresholds, the association between DAO and disability remained significant across both population groups. The absence of a significant subgroup difference suggests that the relationship between DAO and disability is robust across populations when population-specific diagnostic criteria are applied. These findings provide indirect support for the use of ethnically tailored definitions of abdominal obesity and suggest that DAO may serve as a clinically relevant risk phenotype for disability across diverse populations.

The results of age-stratified analyses also demonstrated significant associations between DAO and disability in both younger (<65 years) and older (≥65 years) adults, with no significant difference between age groups. However, one study was not included in the age-based subgroup analysis because participants spanned both age categories and age-specific estimates were unavailable. These findings suggest that the adverse effects of DAO on disability may begin before older age and continue throughout later life. Considering that both reduced muscle strength and abdominal obesity are potentially modifiable conditions, early identification may facilitate the development of interventions targeting these risk factors, although their effectiveness in reducing disability risk requires further investigation. Differences in follow-up duration (4–9 years) may also have contributed to variation in effect estimates across studies, as the impact of DAO is likely to accumulate over time [10,11]. Future studies reporting age-stratified estimates are needed to further clarify whether the association between DAO and disability differs across age groups.

### Limitations

This study contributes to the growing body of longitudinal evidence on the relationship between DAO and disability. Nevertheless, several limitations should be acknowledged. First, differences in DAO definitions, disability measures, follow-up duration, and participant characteristics may have affected the comparability of findings across studies. In addition, although efforts were made to avoid double counting by carefully reviewing cohort characteristics and retaining only one eligible OR estimate from potentially related publications, the possibility of participant overlap among studies conducted in similar settings cannot be completely excluded. Second, because all included studies employed observational cohort designs, the findings should be interpreted as evidence of an association rather than causation. Although important covariates were adjusted for in most studies, the influence of unmeasured or residual confounding factors cannot be excluded. Third, variations in the definitions of both DAO and disability may have influenced the magnitude of the observed associations across studies. In addition, the relatively small number of included studies limited the ability to conduct more detailed subgroup analyses and precluded a formal assessment of publication bias. Furthermore, some subgroup analyses included only one or two studies; therefore, these subgroup findings should be interpreted with caution and require confirmation in future prospective cohort studies. Despite these limitations, the findings were generally consistent across studies and sensitivity analyses. The results suggest that DAO may help identify middle-aged and older adults at increased risk of disability. From a rehabilitation perspective, interventions targeting both muscle weakness and abdominal obesity represent promising strategies for maintaining physical function and supporting independent living; however, their effectiveness in preventing or delaying disability requires confirmation in future prospective intervention studies.

## 5. Conclusions

DAO was associated with increased odds of disability in middle-aged and older adults. Individuals with DAO were more likely to develop disability than those without DAO. These findings highlight the importance of early identification of DAO. In practice, simple assessments of muscle strength and abdominal obesity can be incorporated into routine clinical and community-based evaluations to facilitate the early identification of individuals at increased risk of disability. Interventions such as resistance training, regular physical activity, and appropriate nutritional support represent promising strategies for addressing muscle weakness and abdominal obesity; however, their effectiveness in preventing or delaying disability requires confirmation in future prospective intervention studies. Future research should adopt standardized definitions of DAO and evaluate targeted interventions to better understand their potential role in disability prevention. These findings may also help inform rehabilitation assessment and intervention planning.

## Figures and Tables

**Table 1:** Characteristics of the studies included in the systematic review of the association between dynapenic abdominal obesity and disability.

No.	First Author (Year)	DAO Criteria	Population	Disability Criteria	Sample Size	Country	Sex	Age (Range/Mean)	Follow Up	OR	HR
										(95% CI)	(95% CI)
1	Qian et al. [13]	handgrip strength	Community-dwelling	Functional disability (CHARLS questionnaire)	7881	China	Both	50–63	4 years	2.08 (1.57–2.75)	None
		<28 kg men									
		<18 kg women									
		waist circumference									
		≥90 cm men									
		≥85 cm women									
2	Rossi et al. [14]	handgrip strength	Community-dwelling	IADL decline	846	Italy	Both	65–95	9 years	2.1 (1.14–3.88)	None
		<33 kg men									
		<19 kg women									
		waist circumference									
		>99 cm men									
		>95 cm women									
3	Rossi et al. [15]	handgrip strength	Community-dwelling	ADL limitation	274	Italy	Both	68–78	5.5 years	None	3.39 (1.91–6.02)
		<20.58 kg men									
		<11.66 kg women									
		waist circumference									
		>100 cm men									
		>87 cm women									
4	Smith et al. [16]	handgrip strength	Community-dwelling	Self-reported ADL disability (TILDA dataset)	4471	Ireland	Both	62.3 (average)	4 years	2.15 (1.17–3.93)	None
		<26 kg men									
		<16 kg women									
		waist circumference									
		>102 cm men									
		>88 cm women									
5	Smith et al. [17]	handgrip strength	Community-dwelling	ADL limitation	20,198	China (93%)	Both	56–82	4 years	2.08 (1.37–3.17)	None
		<26 kg men									
		<16 kg women									
		waist circumference									
		>102 cm men									
		>88 cm women									

**Table 2:** Quality assessment of included studies using the Newcastle–Ottawa Scale (NOS).

NOS	Selection				Comparability	Outcome			Total Score	Risk
Articles [Ref]	Representativeness of the Exposed	Selection of the Non-Exposed	Ascertainment of Exposure	Outcome of Interest Was Not Present at Start of Study	Comparability of Cohorts on the Basis of the Design	Assessment of Outcome	Follow-Up Long Enough	Adequacy of Follow-Up of Cohorts		
Qian et al. [13]	1	1	1	1	1	1	0	1	7	Low
Rossi et al. [14]	1	0	1	1	1	1	1	0.5	6.5	Moderate
Rossi et al. [15]	1	0	1	0	0.5	0	0	1	3.5	High risk
Smith et al. [16]	1	1	1	1	0	0	0	0.5	4.5	Moderate
Smith et al. [17]	1	1	0	1	1	0	1	0	5	Moderate

**Table 3:** Leave-one-out sensitivity analysis results.

Omitted Study	OR (95% CI)	*I*^2^ (%)
None	2.13 (1.74–2.60)	0
Qian et al. [13]	2.18 (1.63–2.93)	0
Rossi et al. [14]	2.09 (1.68–2.58)	0
Smith et al. [16]	2.12 (1.72–2.62)	0
Smith et al. [17]	2.14 (1.70–2.69)	0

**Table 4:** Certainty of evidence for the association between DAO and disability.

Outcome	Study Design	Risk of Bias	Inconsistency	Indirectness	Imprecision	Publication Bias	Other Considerations	Effect Estimate (95% CI)	Certainty of Evidence
Disability	Prospective cohort studies	Not serious	Not serious (*I*^2^ = 0%)	Not serious	Not serious (n = 33,670)	Not assessed (<10 studies)	Large effect size	2.13 (1.74–2.60)	⨁⨁◯◯ (low)

GRADE certainty levels: ⨁⨁⨁⨁ = high; ⨁⨁⨁◯ = moderate; ⨁⨁◯◯ = low; ⨁◯◯◯ = very low.

## Data Availability

No new data were created or analyzed in this study. Data sharing is not applicable to this article.

## Supplementary Materials

The following supporting information can be downloaded at https://www.mdpi.com/article/10.3390/healthcare14142125/s1, Table S1: PRISMA 2020 Checklist; Table S2: Complete electronic search strategies used in the systematic review and meta-analysis; Table S3: Adjusted covariates included in each study.
